# Reference Interval for the Axis-Shield Clinical Chemistry Heparin-Binding Protein Assay

**DOI:** 10.3390/diagnostics12081930

**Published:** 2022-08-10

**Authors:** Sumi Yoon, Mina Hur, Hanah Kim, Hee-Won Moon, Yeo-Min Yun

**Affiliations:** 1 Department of Laboratory Medicine, Chung-Ang University College of Medicine, Seoul 06973, Korea; 2 Department of Laboratory Medicine, Konkuk University School of Medicine, Seoul 05030, Korea

**Keywords:** heparin-binding protein, reference interval, analytical performance, Axis-Shield clinical chemistry heparin-binding protein assay

## Abstract

The newly developed Axis-Shield clinical chemistry heparin-binding protein (HBP) assay (Axis-Shield Diagnostics Ltd., Dundee, Scotland) can be applied to fully automated platforms. We aimed to establish a reference interval (RI) of HBP using the Axis-Shield HBP assay, and to evaluate the analytical performance of this assay. An RI was established in 212 sodium citrated plasma samples using the non-parametric method (2.5th and 97.5th percentiles). Precision, linearity, and carry-over were evaluated according to the Clinical and Laboratory Standards Institute guidelines. The RI of HBP was between 5.3 ng/mL and 171.0 ng/mL, which could be applied regardless of gender and age. Percentage coefficients of variations (%CVs) of repeatability and within-laboratory precision were 4.9% and 6.3%, respectively, for low-concentration control and 1.6% and 3.0%, respectively, for high-concentration control. The linearity was excellent (coefficient of determination (R^2^) = 0.99), and the carry-over rate was negligible (0.05%). This is the first study to establish an RI of HBP using the newly developed and fully automated Axis-Shield HBP assay. The Axis-Shield HBP assay showed an acceptable level of analytical performance and could be used to measure HBP concentrations effectively in routine clinical practice. Further studies are awaited to evaluate the clinical utility of HBP using this automated assay.

## 1. Introduction

Sepsis is a major health problem that increases mortality and critical illness, and it is important to identify patients with sepsis as early as possible [1,2]. According to the Third International Consensus Definition for Sepsis and Septic Shock (Sepsis-3), the sequential (sepsis-related) organ failure assessment (SOFA) scoring system is used to identify life-threatening organ dysfunction in sepsis [1]. However, the SOFA scoring system requires clinical judgement, such as the requirement for adrenergic support and Glasgow coma scale scores, which can be subjective and different across institutions [1]. To identify patients with sepsis early, it is necessary to supplement or replace the SOFA scoring system using objective biomarkers [2]. Numerous biomarkers for sepsis have been evaluated, but none are routinely used in clinical practice [3].

Heparin-binding protein (HBP), also known as azurocidin or CAP37 (cationic antimicrobial protein of molecular mass 37 kDa), is released from the azurophilic granules of activated neutrophils and it has multiple functions, such as antimicrobial activity, regulation of monocyte/macrophage, and increased endothelial permeability [2,4,5,6,7]. HBP is a promising biomarker for identifying patients with sepsis [2,8]. For sepsis identification, a clinical cut-off value of 28.1 ng/mL was suggested, which was measured by the enzyme-linked immunosorbent assay (ELISA) [8]. HBP is also associated with other critically ill conditions, including acute kidney injury (AKI), acute respiratory distress syndrome (ARDS), and acute bacterial meningitis [2,7,8,9,10,11,12,13,14,15,16]. In addition, a recent study has reported that HBP concentration increased prior to organ dysfunction in patients with severe coronavirus disease (COVID-19) [17].

In previous studies, HBP concentration has been measured using a non-automated ELISA [7,8,9,10,11,12,13,14,15,16,17]. An ELISA is a simple and easy procedure to perform, and shows high sensitivity and specificity; it is, however, labor-intensive, has a high possibility of false positive and false negative, and shows antibody instability [18]. Many ELISA kits developed to measure HBP concentration have been used for research purposes, not for clinical use [7,8,9,10,11,12,13,14,15,16,17]. Moreover, to the best of our knowledge, the reference interval (RI) of HBP using an ELISA has not been established.

Recently, an assay for measuring HBP concentration, the Axis-Shield clinical chemistry HBP assay (Axis-Shield HBP assay; Axis-Shield Diagnostics Ltd., Dundee, Scotland), was developed. It is the first assay that can be applied to fully automated platforms and is based on latex immunoturbidimetry, not an ELISA [19]. To the best of our knowledge, no studies have been conducted using the fully automated Axis-Shield HBP assay. The RI of HBP has not been established using the Axis-Shield HBP assay, and the analytical performance of this assay has also not been evaluated. Considering that the principle of the Axis-Shield HBP assay is different from that of the ELISA, it is important to establish an RI of HBP using the Axis-Shield HBP assay and to compare the HBP concentrations obtained when using the Axis-Shield HBP assay versus the ELISA. In this study, we aimed to establish an RI of HBP using the Axis-Shield HBP assay. We also evaluated its analytical performance according to the Clinical and Laboratory Standards Institute (CLSI) guidelines for the first time.

## 2. Materials and Methods

### 2.1. Study Population

A total of 212 blood samples were collected from 212 apparently healthy Korean adults who visited the Konkuk University Medical Center (KUMC), Seoul, Korea, for a general medical check-up in December 2017. This study protocol was approved by the Institution Review Board of KUMC (KUH1200033 and 10 December 2013) before recruiting the first sample. Informed consent from the subjects was not required because residual samples were collected after performing the requested test. This study required neither study-intended blood sampling nor other interventions. The healthy Korean adults were included on the basis of their physical and laboratory findings determined by a medical chart review according to the CLSI guidelines [20]. They were considered healthy without any evidence of medical problems, especially inflammation, infection, or sepsis. The individuals who showed abnormal laboratory findings as follows were excluded prior to reaching 212 individuals: white blood cells, <4.0 or >10.0 × 10^9^/L; C-reactive protein, >0.3 mg/dL. Sodium citrated plasma samples were prepared and stored at –80 °C until use. The characteristics of the study population are presented in Table 1.

### 2.2. Axis-Shield Clinical Chemistry HBP Assay

The Axis-Shield HBP assay measured HBP concentrations based on a turbidimetric reaction between HBP and the avian HBP polyclonals. The agglutination between HBP and the HBP antibody bound to polystyrene particles is detected as an absorbance change on an automated clinical chemistry analyzer. The magnitude of the absorbance change is proportional to the HBP concentration in the sample. Only sodium citrated plasma samples are suitable for this assay, which can be applied to many chemistry analyzers [19]. In this study, the Axis-Shield HBP assay was performed using a Toshiba 200FR NEO (Toshiba Medical System Co., Tochigi-ken, Japan), according to the manufacturer’s instructions.

For quality control, the Axis-Shield HBP assay uses the Axis-Shield clinical chemistry HBP controls (Axis-Shield Diagnostics Ltd.), which are recommended to be run daily in duplicate. The 95th percentile reference limit suggested by the manufacturer was 21.44 ng/mL (90% confidence interval (CI), 19.3–23.5 ng/mL), which was established in 53 individuals (19 men, 28 women, and 6 normal samples with unknown demographic details). According to the manufacturer’s instructions, the limit of detection was determined to be 8.0 ng/mL. Percentage coefficients of variations (%CVs) of repeatability and within-laboratory precision were 7% or less for all measured HBP concentrations. The analytical measurement range and linearity was demonstrated from 8.4 to 337.0 ng/mL, and the carry-over was not observed. This assay showed good correlation with a commercially available ELISA in the comparison study [19].

### 2.3. Statistical Analysis

An RI of HBP was established according to the CLSI EP28-A3c guidelines [20]. The distribution of HBP concentration was examined for normality using the Kolmogorov–Smirnov Z-test, and the outliers were checked and excluded using the Dixon–Reed test [21]. The RI was determined using the non-parametric method (2.5th and 97.5th percentiles). The 90% CI was also calculated non-parametrically for each reference limit [20]. The study population was divided into two groups depending on gender (men, *n* = 93; women, *n* = 119) and six groups depending on age (years; 19–29, *n* = 45; 30–39, *n* = 40; 40–49, *n* = 37; 50–59, *n* = 38; 60–69, *n* = 34; and 70–79, *n* = 18). The median value of HBP concentration was compared between genders using the Mann–Whitney U test and between ages using the Kruskal–Wallis test.

To evaluate precision, the Axis-Shield HBP controls were analyzed according to the CLSI EP05-A3 guidelines [22]. After preliminary evaluation, two different controls (low- and high-concentrations) were analyzed in duplicate per run, two runs per day, for 20 days (2 × 2 × 20 experiment design for each control). Repeatability and within-laboratory precision were evaluated using the analysis of variance (ANOVA) and expressed as standard deviation (SD) and %CV. %CV were interpreted as follows: %CV ≤10%, excellent; 10–20%, good; 20–30%, acceptable; >30%, poor [23,24]. The linearity was evaluated according to the CLSI EP06-A guidelines [25]. A calibrator of 334.0 ng/mL was diluted to five different concentrations (0%, 25%, 50%, 75%, and 100%). The serially diluted samples were analyzed in quadruplicate at each concentration. The linearity was determined using linear regression analysis with 95% CI. The recoveries were also calculated as a percentage and 100% ± 10% was considered acceptable. The carry-over was evaluated using low- and high-concentration controls, which were analyzed in quadruplicate, respectively, according to the CLSI EP10-A3-AMD guidelines [26]. The equation for calculating the carry-over rate was as follows: %carry-over = [L1 − (L3 + L4)/2 × 100]/[(H2 + H3)/2 − (L3 + L4)/2]. The calculated carry-over rate of less than 1.0% was considered acceptable [27].

Statistical analyses were performed using MedCalc Statistical Software (version 20.109; MedCalc Software, Ostend, Belgium) and Microsoft Excel Software (version 2016; Microsoft Corporation, Redmond, WA, USA). Two-sided *p* < 0.05 was considered statistically significant.

## 3. Results

### 3.1. Reference Interval

**Figure 1:**
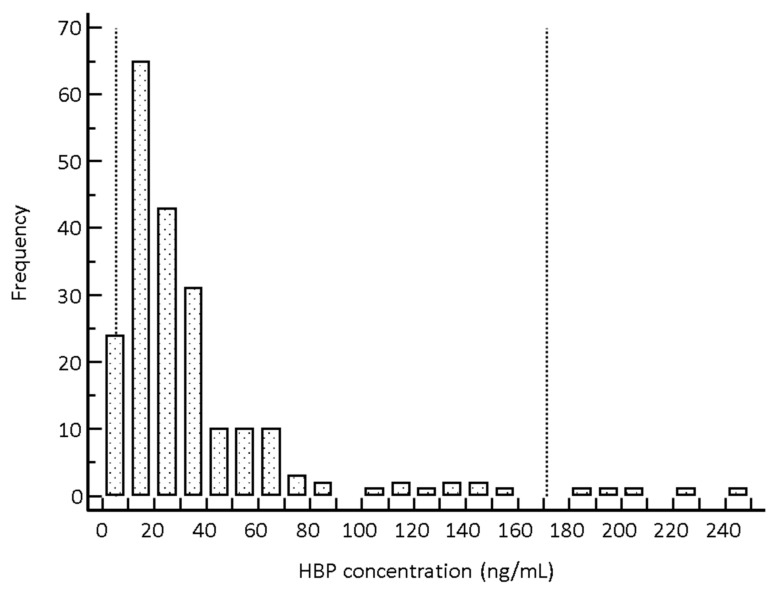
Distribution of HBP concentration measured by the Axis-Shield HBP assay in total individuals (*n* = 212). Dotted lines represent the 2.5th and 97.5th percentile reference limits. Abbreviations: HBP, heparin-binding protein; n, number.

The HBP concentrations showed a right-skewed distribution with no outlier (Figure 1). In all individuals (*n* = 212), the lower (2.5th percentile) and upper (97.5th percentile) reference limits of HBP were 5.3 ng/mL (90% CI, 4.4–6.3 ng/mL) and 171.0 ng/mL (90% CI, 137.5–227.8 ng/mL), respectively. The median value of HBP concentration was 23.5 ng/mL (95% CI, 20.5–25.8 ng/mL) in all individuals. The lower and upper reference limits of HBP in men (*n* = 93) were 5.6 and 195.7 ng/mL, respectively, and 5.3 and 148.9 ng/mL in women (*n* = 119), respectively. There were no statistically significant differences in HBP concentration between men and women (median, 25.5 vs. 21.1 ng/mL, *p* = 0.12). The median value of HBP concentration in each age group was: 19–29, 20.9 ng/mL; 30–39, 21.4 ng/mL; 40–49, 23.5 ng/mL; 50–59, 21.7 ng/mL; 60–69, 25.2 ng/mL; and 70–79, 28.3 ng/mL. There were no statistically significant differences in HBP concentration between ages (*p* = 0.85).

### 3.2. Analytical Performance

**Figure 2:**
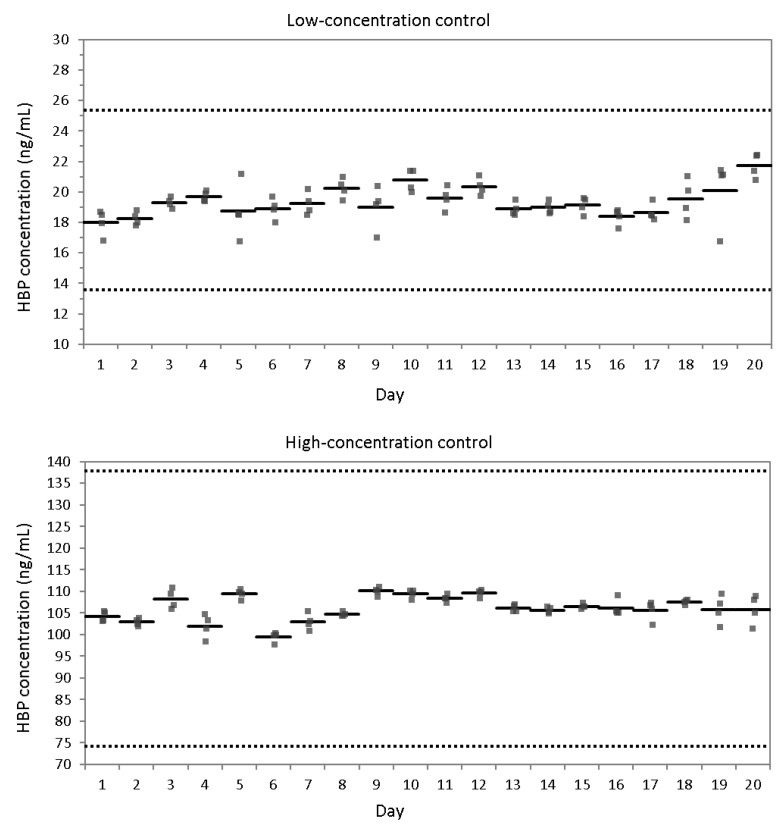
The analytical measurements of precision evaluation for low- and high-concentration controls. Solid bars are mean values of each day, and dotted lines are acceptable %CV limits of 30%. Abbreviations: %CV, percentage coefficient of variations; HBP, heparin-binding protein.

**Figure 3:**
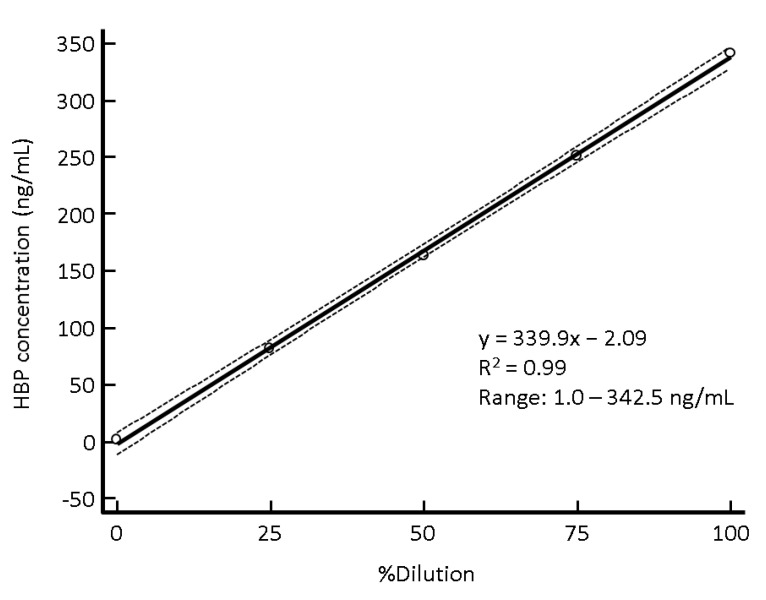
Linearity of the Axis-Shield HBP assay. The solid line indicates the regression equation, and the dotted lines indicate the 95% CI for the regression equation. Abbreviations: CI, confidence interval; HBP, heparin-binding protein.

The analytical measurements for evaluating precision ranged from 16.7 to 21.2 ng/mL for low-concentration control and from 98.3 to 110.9 ng/mL for high-concentration control. The %CVs of repeatability for low- and high-concentration controls were 4.9% and 1.6%, respectively. The %CVs of within-laboratory precision for low- and high-concentration controls were 6.3% and 3.0%, respectively. All %CVs of repeatability and within-laboratory precision were excellent for low- and high-concentration controls. There were no apparent outliers or drift capable of distorting the precision analysis (Figure 2). The analytical measurements for evaluating linearity ranged from 1.0 to 342.5 ng/mL, and the linearity was excellent with the coefficient of determination (R^2^) = 0.99 (Figure 3). Percentage recoveries of serially diluted samples were acceptable, ranging from 96.2% to 100.5%. The carry-over rate was negligible with 0.05%.

## 4. Discussion

The newly developed Axis-Shield HBP assay is a fully automated assay that has not been evaluated so far. Many previous studies have reported measuring HBP concentration using the ELISA [7,8,9,10,11,12,13,14,15,16,17]. To the best of our knowledge, neither the clinical cut-off value nor the RI of HBP using the Axis-Shield HBP assay have been established. This is the first study worldwide, which established an RI of HBP using the fully automated Axis-Shield HBP assay and evaluated its analytical performance based on the CLSI guidelines.

The established RI for HBP in Korean adults was from 5.3 to 171.0 ng/mL, which could be applied regardless of gender and age. The reference limit as the 95th percentile established in our study population was 131.9 ng/mL, which was much higher than the reference limit suggested by the manufacturer. HBP concentration could increase not only in patients with infection and sepsis but also in patients with non-infectious diseases such as ST-segment elevation myocardial infarction [28]. Although we included apparently healthy individuals, some asymptomatic patients with non-infectious diseases may have been included in this study and may have influenced the establishment of a high reference limit. However, we conducted this study on individuals without critical illness that could affect HBP concentrations through a thorough medical chart review of clinical and laboratory data [2,7,8,9,10,11,12,13,14,15,16,28]. In addition, the reference limit suggested by the manufacturer was established in a small cohort of 53 samples without information on ethnicity or skewness of the distribution [19]. According to the manufacturer’s instructions, the Axis-Shield HBP assay was developed for in vitro diagnostic use, and each laboratory needs to evaluate and establish its own RI of HBP in large cohorts [19]. In this study, there were some skewed populations with high HBP concentrations even in healthy individuals (Figure 1). This result implies that HBP concentration may be high without sepsis or other known ill conditions. Several biomarkers, including biologically active adrenomedullin and proenkephalin, have been reported that could be objective and useful markers to predict severity, organ failure, and mortality in septic patients [29,30]. Since the concentration of these biomarkers vary among individuals, it is mandatory to understand the biological variation (BV) of a biomarker in healthy individuals [31,32]. However, the BV of HBP has not been assessed [33]. Further prospective studies on the RI and BV of HBP in large cohorts are awaited.

Compared with a clinical cut-off value of 28.1 ng/mL suggested in a previous study using the ELISA (manufactured by Axis-Shield), approximately 40% of our study population had a higher HBP concentration [8]. In the previous study, all individuals in the healthy group had an HBP concentration of <10 ng/mL, but the healthy group consisted of a small number of 56 individuals [8]. The median value of HBP concentration in our study population was 23.5 ng/mL, which was not significantly different from the suggested cut-off value. The clinical cut-off values for sepsis differed between methods with the same ELISA principle [7,8,9]. There is no consensus on the universal and disease-specific cut-off values of HBP, and the cut-off values suggested in previous studies were based on a small number of samples [7,8,9,10,11,12,13,14,15,16,17]. The previous studies were conducted to compare the difference in HBP concentrations between small groups using the ELISA, not to establish the RI of HBP [7,8,9,10,11,12,13,14,15,16,17]. We focused only on establishing the RI of HBP using the Axis-Shield HBP assay. It is necessary to compare the RI established in this study with the HBP concentration measured by the Axis-Shield HBP assay in septic patients. In addition, it should be considered that HBP concentration could increase in non-infectious disease [28]. A clinical cut-off value is appropriate in large-scale studies, but there are no studies yet on the cut-off value of HBP. It is too early to determine the universal cut-off value, as previous studies have used only small cohorts. Even in patients with sepsis or septic shock, the concentration of procalcitonin, a well-known biomarker for sepsis, might be lower than the clinical cut-off value of 0.5 ng/mL [34]. Sepsis cannot be identified with a single biomarker, and a multi-marker approach is emphasized [35,36].

In this study, the Axis-Shield HBP assay showed excellent repeatability and within-laboratory precision with less than 10% CV. It also showed excellent linearity and an acceptable carry-over rate that had less than 1.0% cut-off limit. These results are similar to the analytical performance provided by the manufacturer [19]. This indicates that the Axis-Shield HBP assay is acceptable for use in measuring HBP concentration in clinical laboratories.

The strength of this study is to provide the fundamental data of the Axis-Shield HBP assay for further studies and clinical use. On the other hand, this study has several limitations. First, this study was conducted on individuals who were considered healthy only by a medical chart review among those who visited the KUMC for a general medical check-up. Individuals with critical illnesses, such as AKI and ARDS that could affect the HBP concentration were excluded. However, factors such as underlying health conditions and drug use could not be considered in establishing the RI of HBP, as undisclosed health conditions of the subjects could not be checked. Second, the number of individuals in each gender and age was less than 120 in this study. The CLSI guidelines have recommended that at least 120 individuals for each gender or other subclass are required to determine whether to partition RIs by calculating the statistic z-value [20]. Inevitably, the median value of HBP concentration for each gender and age was compared in this study. Although the statistic z-value was not calculated, it would be acceptable to apply a single RI regardless of gender and age. Third, we established the RI only for Korean adults. Previous studies have reported that HBP concentrations of urine and sputum in children are associated with urinary tract infections and pulmonary inflammation, respectively; HBP could be a promising biomarker even in children [37,38,39]. Although there were no statistically significant differences in HBP concentration between ages in Korean adults in our study, it is necessary to evaluate the age-specific RI in large cohorts, including children. Finally, we did not compare the HBP concentration between the Axis-Shield HBP assay and the ELISA. Procalcitonin has also been reported to show a modest bias between different reagents and analyzers [40]. Considering that the principle is different between the Axis-Shield HBP assay and the ELISA, the HBP concentration could show a bias between them. It is necessary to further evaluate the correlation between the Axis-Shied HBP assay and the ELISA for HBP. Even though the two methods (immunoturbidimetry and ELISA) show good agreement, there may be a difference in values [41]. If HBP is to be used as a biomarker with a critical threshold for sepsis or other infections, standardization should be achieved between methods with different principles, and even between different reagents and analyzers within the same principle.

In conclusion, we established an RI of HBP using the fully automated Axis-Shield HBP assay, and the RI could be applied regardless of gender and age. The Axis-Shield HBP assay showed an acceptable level of analytical performance according to the CLSI guidelines. It could be useful to measure HBP concentrations in routine clinical practice. Further studies are awaited to evaluate clinical utility of HBP using this automated assay in various critically ill patients. It should be performed after comparing the HBP concentrations of the automated assay and the ELSIA.

## Figures and Tables

**Table 1:** Characteristics of the study population for establishing an RI of HBP.

Characteristic	Total	Men	Women
n (%)	212 (100.0)	93 (43.9)	119 (56.1)
Age (yrs), (median, range)	45 (19–79)	44 (19–78)	45 (19–79)
CBC (median, IQR)			
WBC (×10^9^/L)	6.76 (5.87–7.93)	6.96 (5.99–8.01)	6.61 (5.78–7.93)
Hb (g/L)	141 (131–149)	150 (145–158)	132 (128–140)
Platelet (×10^9^/L)	244 (216–285)	231 (206–279)	249 (219–291)
CRP (mg/dL), (median, IQR)	0.05 (0.03–0.10)	0.06 (0.03–0.11)	0.04 (0.03–0.10)
HBP (ng/mL), (median, IQR)	23.5 (14.8–38.8)	25.5 (15.8–44.0)	21.1 (13.9–35.7)

Abbreviations: CBC, complete blood counts; CRP, C-reactive protein; Hb, hemoglobin; HBP, heparin-binding protein; IQR, interquartile range; n, number; WBC, white blood cell; yrs, years.

## Data Availability

The data presented in this study are available from the corresponding author on reasonable request.

## References

[1] Singer M., Deutschman C.S., Seymour C.W., Shankar-Hari M., Annane D., Bauer M., Bellomo R., Bernard G.R., Chiche J.D., Coopersmith C.M. (2016). The third international consensus definitions for sepsis and septic shock (Sepsis-3). JAMA.

[2] Fisher J., Linder A. (2017). Heparin-binding protein: A key player in the pathophysiology of organ dysfunction in sepsis. J. Intern. Med..

[3] Pierrakos C., Vincent J.L. (2010). Sepsis biomarkers: A review. Crit. Care.

[4] Pereira H.A. (1995). CAP37, a neutrophil-derived multifunctional inflammatory mediator. J. Leukoc. Biol..

[5] Tapper H., Karlsson A., Mörgelin M., Flodgaard H., Herwald H. (2002). Secretion of heparin-binding protein from human neutrophils is determined by its localization in azurophilic granules and secretory vesicles. Blood.

[6] Linder A., Soehnlein O., Akesson P. (2010). Roles of heparin-binding protein in bacterial infections. J. Innate Immun..

[7] Linder A., Arnold R., Boyd J.H., Zindovic M., Zindovic I., Lange A., Paulsson M., Nyberg P., Russell J.A., Pritchard D. (2015). Heparin-binding protein measurement improves the prediction of severe infection with organ dysfunction in the emergency department. Crit. Care Med..

[8] Zhou Y., Liu Z., Huang J., Li G., Li F., Cheng Y., Xie X., Zhang J. (2019). Usefulness of the heparin-binding protein level to diagnose sepsis and septic shock according to Sepsis-3 compared with procalcitonin and C reactive protein: A prospective cohort study in China. BMJ Open.

[9] Linder A., Christensson B., Herwald H., Björck L., Akesson P. (2009). Heparin-binding protein: An early marker of circulatory failure in sepsis. Clin. Infect. Dis..

[10] Chew M.S., Linder A., Santen S., Ersson A., Herwald H., Thorlacius H. (2012). Increased plasma levels of heparin-binding protein in patients with shock: A prospective, cohort study. Inflamm. Res..

[11] Linder A., Åkesson P., Inghammar M., Treutiger C.J., Linner A., Sunden-Cullberg J. (2012). Elevated plasma levels of heparin-binding protein in intensive care unit patients with severe sepsis and septic shock. Crit. Care.

[12] Lin Q., Shen J., Shen L., Zhang Z., Fu F. (2013). Increased plasma levels of heparin-binding protein in patients with acute respiratory distress syndrome. Crit. Care.

[13] Tverring J., Vaara S.T., Fisher J., Poukkanen M., Pettilä V., Linder A., FINNAKI Study Group (2017). Heparin-binding protein (HBP) improves prediction of sepsis-related acute kidney injury. Ann. Intensive Care.

[14] Tydén J., Herwald H., Hultin M., Walldén J., Johansson J. (2017). Heparin-binding protein as a biomarker of acute kidney injury in critical illness. Acta Anaesthesiol. Scand..

[15] Kandil M., Khalil G., El-Attar E., Shehata G., Hassan S. (2018). Accuracy of heparin binding protein: As a new marker in prediction of acute bacterial meningitis. Braz. J. Microbiol..

[16] Kahn F., Tverring J., Mellhammar L., Wetterberg N., Bläckberg A., Studahl E., Hadorn N., Kahn R., Nueesch S., Jent P. (2019). Heparin-binding protein as a prognostic biomarker of sepsis and disease severity at the emergency department. Shock.

[17] Mellhammar L., Thelaus L., Elén S., Fisher J., Linder A. (2021). Heparin binding protein in severe COVID-19-A prospective observational cohort study. PLoS ONE.

[18] Sakamoto S., Putalun W., Vimolmangkang S., Phoolcharoen W., Shoyama Y., Tanaka H., Morimoto S. (2018). Enzyme-linked immunosorbent assay for the quantitative/qualitative analysis of plant secondary metabolites. J. Nat. Med..

[19] Axis-Shield Clinical Chemistry Heparin Binding Protein Reagent Kit. https://www.axis-shield.com//wp-content/uploads/2019/12/FHHBP101_-ASD-CC-HBP_Reagent-IFU_RPBL1302-R2.pdf.

[20] (2010). Defining, Establishing, and Verifying Reference Intervals in the Clinical Laboratory; Approved Guideline—Third Edition.

[21] Reed A.H., Henry R.J., Mason W.B. (1971). Influence of statistical method used on the resulting estimate of normal range. Clin. Chem..

[22] (2014). Evaluation of Precision of Quantitative Measurement Procedures; Approved Guideline—Third Edition.

[23] Barnhart H.X., Barboriak D.P. (2009). Applications of the repeatability of quantitative imaging biomarkers: A review of statistical analysis of repeat data sets. Transl. Oncol..

[24] Lecler A., Savatovsky J., Balvay D., Zmuda M., Sadik J.C., Galatoire O., Charbonneau F., Bergès O., Picard H., Fournier L. (2017). Repeatability of apparent diffusion coefficient and intravoxel incoherent motion parameters at 3.0 Tesla in orbital lesions. Eur. Radiol..

[25] (2003). Evaluation of the Linearity of Quantitative Measurement Prodedures: A Statistical Approach; Approved Guideline.

[26] (2014). Preliminary Evaluation of Quantitative Clinical Laboratory Measurement Procedures; Approved Guideline—Third Edition.

[27] Broughton P.M. (1984). Carry-over in automatic analysers. J. Automat. Chem..

[28] Yang Y., Liu G., He Q., Shen J., Xu L., Zhu P., Zhao M. (2019). A Promising candidate: Heparin-binding protein steps onto the stage of sepsis prediction. J. Immunol. Res..

[29] Kim H., Hur M., Struck J., Bergmann A., Di Somma S. (2019). Circulating biologically active adrenomedullin predicts organ failure and mortality in sepsis. Ann. Lab. Med..

[30] Kim H., Hur M., Struck J., Bergmann A., Di Somma S. (2020). Proenkephalin predicts organ failure, renal replacement therapy, and mortality in patients with sepsis. Ann. Lab. Med..

[31] Wu A.H. (2013). Biological and analytical variation of clinical biomarker testing: Implications for biomarker-guided therapy. Curr. Heart Fail. Rep..

[32] Aziz N., Detels R., Quint J.J., Gjertson D., Ryner T., Butch A.W. (2019). Biological variation of immunological blood biomarkers in healthy individuals and quality goals for biomarker tests. BMC Immunol..

[33] European Federation of Clinical Chemistry and Laboratory Medicine (EFLM) Biological Variation Database. https://biologicalvariation.eu.

[34] Park M., Hur M., Kim H., Lee C.H., Lee J.H., Kim H.W., Nam M. (2022). Prognostic utility of procalcitonin, presepsin, and the VACO index for predicting 30-day mortality in hospitalized COVID-19 patients. Ann. Lab. Med..

[35] Hur M., Kim H., Lee S., Cristofano F., Magrini L., Marino R., Gori C.S., Bongiovanni C., Zancla B., Cardelli P. (2014). Diagnostic and prognostic utilities of multimarkers approach using procalcitonin, B-type natriuretic peptide, and neutrophil gelatinase-associated lipocalin in critically ill patients with suspected sepsis. BMC Infect. Dis..

[36] Kim H., Hur M., Moon H.W., Yun Y.M., Di Somma S., GREAT Network (2017). Multi-marker approach using procalcitonin, presepsin, galectin-3, and soluble suppression of tumorigenicity 2 for the prediction of mortality in sepsis. Ann. Intensive Care.

[37] Kjölvmark C., Akesson P., Linder A. (2012). Elevated urine levels of heparin-binding protein in children with urinary tract infection. Pediatr. Nephrol..

[38] El-Sabbagh A.M., Abd Elmagid D.S. (2017). Heparin-binding protein as an immunological marker in children urinary tract infection. Egypt. J. Med. Microbiol..

[39] Hovold G., Palmcrantz V., Kahn F., Egesten A., Påhlman L.I. (2018). Heparin-binding protein in sputum as a marker of pulmonary inflammation, lung function, and bacterial load in children with cystic fibrosis. BMC Pulm. Med..

[40] Dipalo M., Guido L., Micca G., Pittalis S., Locatelli M., Motta A., Bianchi V., Callegari T., Aloe R., Da Rin G. (2015). Multicenter comparison of automated procalcitonin immunoassays. Pract. Lab. Med..

[41] Fellahi S., Béraud L., Marlin G., Vigouroux C., Warszawski J., Capeau J., Bastard J.P. (2017). Comparison of two techniques of adiponectin assay, ELISA and immunoturbidimetry: Should we move towards standardization?. Diabetes Metab..

